# A Single Nucleotide ADA Genetic Variant Is Associated to Central Inflammation and Clinical Presentation in MS: Implications for Cladribine Treatment

**DOI:** 10.3390/genes11101152

**Published:** 2020-09-30

**Authors:** Mario Stampanoni Bassi, Fabio Buttari, Ilaria Simonelli, Luana Gilio, Roberto Furlan, Annamaria Finardi, Girolama Alessandra Marfia, Andrea Visconti, Andrea Paolillo, Marianna Storto, Stefano Gambardella, Rosangela Ferese, Marco Salvetti, Antonio Uccelli, Giuseppe Matarese, Diego Centonze, Francesca De Vito

**Affiliations:** 1 Unit of Neurology, IRCCS Neuromed, Via Atinense 18, 86077 Pozzilli (IS), Italy; m.stampanonibassi@gmail.com (M.S.B.); fabio.buttari@gmail.com (F.B.); gilio.luana@gmail.com (L.G.); marfia@med.uniroma2.it (G.A.M.); maristor@yahoo.com (M.S.); stefano.gambardella@neuromed.it (S.G.); ferese.rosangela@gmail.com (R.F.); marco.salvetti@uniroma1.it (M.S.); f.devito.molbio@gmail.com (F.D.V.); 2 Service of Medical Statistics & Information Technology, Fondazione Fatebenefratelli per la Ricerca e la Formazione Sanitaria e Sociale, 00186 Rome, Italy; ilaria.simonelli@afar.it; 3 Neuroimmunology Unit, Institute of Experimental Neurology (INSpe), Division of Neuroscience, San Raffaele Scientific Institute, 20132 Milan, Italy; furlan.roberto@hsr.it (R.F.); finardi.annamaria@hsr.it (A.F.); 4 Multiple Sclerosis Clinical and Research Unit, Department of Systems Medicine, University of Rome “Tor Vergata”, 00133 Rome, Italy; 5 Affiliate of Merck KGaA, Frankfurter St. 250, 64293, Darmstadt, Germany; andrea.visconti@merckgroup.com (A.V.); andrea.paolillo@merckgroup.com (A.P.); 6 Department of Biomolecular Sciences, University of Urbino “Carlo Bo”, 61029 Urbino, Italy; 7 Center for Experimental Neurological Therapies, Sant’Andrea Hospital, Department of Neurosciences, Mental Health and Sensory Organs, Sapienza University of Rome, 00189 Rome, Italy; 8 Department of Neurosciences, Rehabilitation, Ophthalmology, Genetics, Maternal and Child Health Unit and Center of Excellence for Biomedical Research, University of Genova, 16100 Genoa, Italy; auccelli@neurologia.unige.it; 9 IRCCS Ospedale Policlinico San Martino, 16132 Genoa, Italy; 10 Treg Cell Lab, National Research Council (IEOS-CNR), Institute of Experimental Oncology and Endocrinology, 80131 Naples, Italy; giuseppe.matarese@unina.it; 11 Department of Molecular Medicine and Biotechnologies, University of Naples “Federico II”, 80131 Naples, Italy; 12 Laboratory of Synaptic Immunopathology, Department of Systems Medicine, Tor Vergata University, Via Montpellier 1, 00133 Rome, Italy

**Keywords:** ADA, cladribine tablets, IL-10, inflammation, TNF, multiple sclerosis

## Abstract

In multiple sclerosis (MS), activated T and B lymphocytes and microglial cells release various proinflammatory cytokines, promoting neuroinflammation and negatively affecting the course of the disease. The immune response homeostasis is crucially regulated by the activity of the enzyme adenosine deaminase (ADA), as evidenced in patients with genetic ADA deficiency and in those treated with cladribine tablets. We investigated in a group of patients with MS the associations of a single nucleotide polymorphism (SNP) of ADA gene with disease characteristics and cerebrospinal fluid (CSF) inflammation. The SNP rs244072 of the ADA gene was determined in 561 patients with MS. Disease characteristics were assessed at the time of diagnosis; furthermore, in 258 patients, proinflammatory and anti-inflammatory molecules were measured in the CSF. We found a significant association between rs244072 and both clinical characteristics and central inflammation. In C-carriers, significantly enhanced disability and increased CSF levels of TNF, IL-5 and RANTES was observed. In addition, lower CSF levels of the anti-inflammatory cytokine IL-10 were found. Finally, the presence of the C allele was associated with a tendency of increased lymphocyte count. In MS patients, ADA SNP rs244072 is associated with CSF inflammation and disability. The selective targeting of the ADA pathway through cladribine tablet therapy could be effective in MS by acting on a pathogenically relevant biological mechanism.

## 1. Introduction

Multiple sclerosis (MS) is a chronic autoimmune disease characterized by demyelination and the neurodegeneration of the central nervous system (CNS). Autoreactive T and B lymphocytes play a crucial role in disease pathogenesis [1,2]. T cells enter CNS and interact with local immune cells, initiating the inflammatory cascade that amplifies the immune response and leads to both excitotoxic synaptopathy and consequent brain damage [3,4]. Both CD4^+^ and CD8^+^ autoreactive T lymphocytes have been identified in demyelinating lesions [1] and have been related to the severity of axonal damage and inflammation [3]. Additionally, B lymphocytes contribute to MS development and progression, acting as antibody-producing and antigen-presenting cells, as well as releasing soluble mediators involved in tissue damage and in the regulation of the immune response [2]. Pro-inflammatory and anti-inflammatory molecules critically regulate the induction and maintenance of the inflammatory response, ultimately affecting the disease course [5]. Accordingly, proinflammatory cytokines released by lymphocytes and microglial cells alter the blood brain barrier, increasing immune cell extravasation [6], and contributing to demyelinating damage [7].

Targeting T and B lymphocytes constitutes a promising therapeutic approach in MS. Lymphocytes are sensitive to impaired nucleotide metabolism because it affects their entire life, from DNA synthesis and integrity to the inflammatory response. In particular, the altered activity of the enzyme adenosine deaminase (*ADA*) is associated with the toxic accumulation of triphosphorylated deoxyadenosine and lymphocyte depletion, as shown in *ADA* genetic deficiency and in patients treated with cladribine (2-chloro-2′-deoxyadenosine) tablets, a purine deoxynucleoside analogue resistant to the action of *ADA*, approved for the treatment of highly active relapsing–remitting (RR)MS [8,9]. Accumulating research has explored ADA deregulation in many types of autoimmune diseases [10], but few data are available in MS. The activity and the level of *ADA* have been reported to be altered, respectively, in peripheral and central compartments of patients with MS compared to healthy subjects [11,12].

In this study, we investigated, in a large cohort of patients with MS, the association of a single nucleotide polymorphism (SNP) in *ADA* gene and cerebrospinal fluid (CSF) inflammation as well as clinical characteristics. We hypothesize that, between the complete absence of *ADA* and its full expression and function, there could be intermediate conditions related to individual genetic variability which, although not altering the normal immune responses, could influence the pathophysiology of autoimmune diseases and particularly of MS.

## 2. Materials and Methods

### 2.1. MS Patients

A group of 561 consecutive MS patients were enrolled in the study. Patients were admitted to the neurological clinic of the University Hospital Tor Vergata in Rome and Neuromed Research Institute in Pozzilli, Italy, between 2010 and 2017, and diagnosed with MS on the basis of clinical, laboratory and MRI parameters [13]. The Ethics Committees of the University Tor Vergata Hospital in Rome (cod.138-10) and Neuromed Research Institute in Pozzilli, Italy (cod. 06-17) approved the study according to the Declaration of Helsinki. All patients gave written informed consent to participate in the study. During hospitalization, patients underwent clinical evaluation and brain and spine MRI. Clinical characteristics were recorded at the time of diagnosis, including age, sex, expanded disability status score (EDSS), the presence of clinical/radiological disease activity, and disease duration, measured as the interval between disease onset and diagnosis. Patients underwent a 1.5 or 3 Tesla MRI, including dual-echo proton density, fluid-attenuated inversion recovery, T1-weighted spin-echo (SE), T2-weighted fast SE, and contrast-enhanced T1-weighted SE after intravenous gadolinium (Gd) infusion (0.2 mL/kg). Radiological disease activity at the time of diagnosis was defined as the presence of Gd-enhancing (Gd+) lesions at the time of hospitalization.

### 2.2. SNP rs244072 Analysis

SNPs in non-coding regions of *ADA* gene with potential regulatory functions of mRNA expression were identified by using the UCSC Genome Browser [14] (human genome assembly Feb. 2009 GRCh37/hg19). Specifically, we focused on DNase hypersensitive sites containing enhancer-like signatures classified by the ENCODE Data Analysis Center. Then, by RBPmap Version 1.1 [15], we searched consensus sequences for RNA binding proteins (RBPs), which included the identified SNPs with minor allele frequency of 8% or more. Finally, by the functional annotation with DAVID 6.8 [16] and PubMed [17] we selected rs244072 as good candidate for further studies in MS, being the SNP located into putative binding sites for RBPs crucial in lymphocyte activation [18,19,20].

Thus, genotyping for *ADA* SNPs rs244072 was performed in all enrolled patients. A blood sample for the genetic screening was collected from each patient at the time of diagnosis. Genotyping was performed by polymerase chain reaction with the TaqMan method performed using the ABI-Prism 7900HT Sequence Detection System (Applied Biosystems, Foster City, CA, United States).

### 2.3. CSF Collection and Analysis

In 258, MS patients’ CSF concentrations of inflammatory cytokines were analyzed. CSF was collected at the time of diagnosis, during hospitalization, by lumbar puncture (LP). No corticosteroids were administered before LP. Disease modifying therapies were initiated after the confirmed diagnosis when indicated. CSF was stored at −80 °C and then analyzed using a Bio-Plex multiplex cytokine assay (Bio-Rad Laboratories, Hercules, CA, USA). CSF cytokines levels were determined according to a standard curve generated for the specific target and expressed as picograms/milliliter (pg/mL). Samples were analyzed in triplicate. The CSF cytokines analyzed included interleukin (IL)-1β, IL-2, IL-4, IL-5, IL-6, IL-7, IL-8, IL-10, IL-12, IL-13, tumor necrosis factor (TNF) and RANTES.

### 2.4. Blood Samples Collection and WBC Count/Analysis

In an independent cohort of 230 MS patients, we explored the association between *ADA SNP rs244072* and peripheral white blood cells (white blood cells count, WBC; neutrophils; lymphocytes).

Peripheral blood samples were collected by standard venipuncture (EDTA collection tube (Vacutainer^®^, Becton Dickinson, Milan, Italy). For the blood exam we used a hematology analyzer ADVIA 2120i system (Siemens Healthcare Diagnostics Inc., NY, USA) whose technology utilizes peroxidase staining that permits the cytochemical differentiation of myeloid and lymphoid cells. We calculated the neutrophil/lymphocyte ratio (NLR) by dividing the neutrophil number by the lymphocyte number [21].

### 2.5. Statistical Analysis

Shapiro–Wilk test was used to evaluate normality distribution of continuous variables. Data were shown as mean (standard deviation, SD) or median (interquartile range, IQR). Categorical variables were presented as absolute (n) and relative frequency (%). Chi-square or, when necessary, Fisher exact test, were employed to explore the association between categorical variables. Difference in continuous variables between the *ADA* SNP groups was evaluated using nonparametric Mann–Whitney test. The Benjamini–Hochberg (B–H) false discovery rate controlling procedure was applied for multiple testing. A *p* value ≤ 0.05 was considered statistically significant. To show statistically significant differences in CSF cytokines levels and EDSS scores between two categories, a box plot was used. To better show the cytokines levels’ distribution, the values were depicted on logarithmic scale. All analyses were performed using IBM SPSS Statistics for Windows (IBM Corp., Armonk, NY, USA).

## 3. Results

### 3.1. The ADA SNP rs244072 in Patients with MS

**Figure 1:**
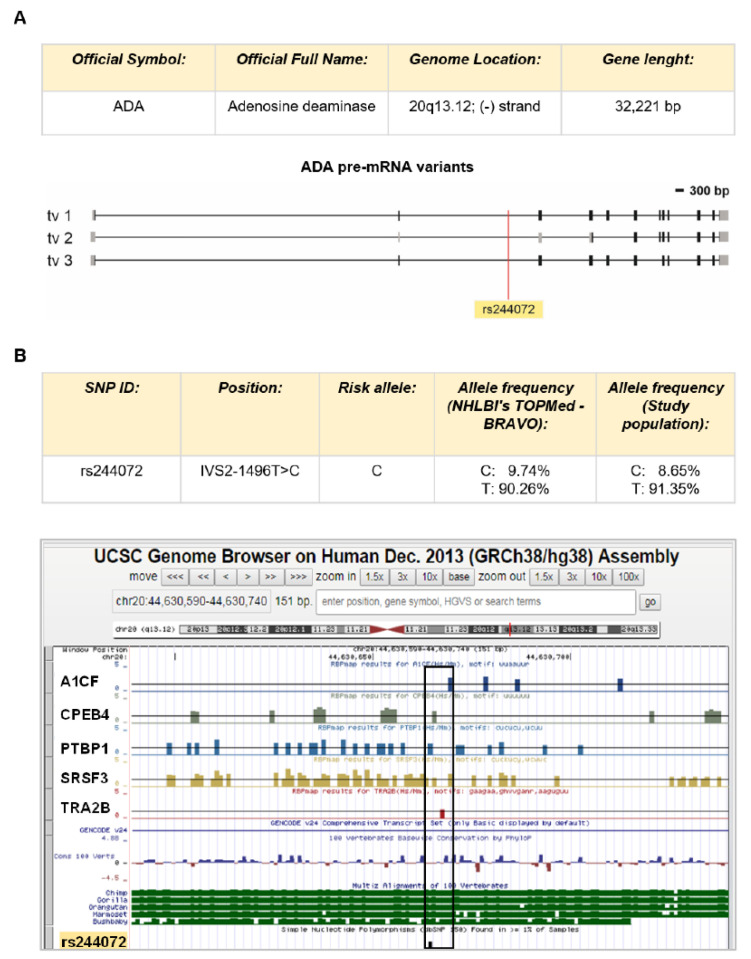
*ADA* gene and SNP rs244072. (**A**) Scheme of *ADA* gene. The *ADA* gene in humans is placed in the minus strand of the chromosome 20 (chr20:44,619,522–44,651,742 in human genome assembly Dec. 2013 GRCh38/hg38). Three splicing variants coding for functional proteins (transcript variant 1 with ID NM_000022 = tv 1, transcript variant 2 with ID NM_001322050 = tv 2, transcript variant 3 with ID NM_00132205 = tv 3) are depicted according to NCBI annotations. Black rectangles represent coding regions in exons. Grey rectangles are untranslated regions in exons. Black lines are introns. “bp” means base pair. SNP rs244072 is located in the second intron of *ADA* gene and in all transcript variants. (**B**) Description of the ADA SNP rs244072. The SNP is 1496 bp upstream the end of second intron (chr20:44630665 in human genome assembly Dec. 2013 GRCh38/hg38). Allele frequencies (C/T) from both European population from NHLBI’s TOPMed-BRAVO database (https://bravo.sph.umich.edu/freeze5/hg38/variant/20-44630665-A-G) and our sample population are reported. Bioinformatic analysis on ±75 bp from rs244072 by using RBPmap revealed that the SNP is included in several RNA binding sites for different regulatory proteins of gene expression. UCSC representation of the RNA binding site is outlined. The black empty rectangle identifies the regulatory regions containing rs244072. RNA binding proteins’ ID are indicated on the left (A1CF = APOBEC1 Complementation Factor; CPEB4 = cytoplasmic polyadenylation element binding protein 4; PTBP1 = Polypyrimidine Tract Binding Protein 1; SRSF3 = Serine and arginine Rich Splicing Factor 3; TRA2B = TRAnsformer 2 β homolog).

By in silico analysis of non-coding regulatory regions of *ADA* gene, we identify the SNP rs244072 as a good candidate to test our hypothesis. This SNP is placed in the second intron of the *ADA* gene (IVS2-1496T>C) inner putative binding sites for five different modulators of mRNA processing, as emerged by RBPmap prediction (Figure 1A). In particular, the rs244072 variants could influence *ADA* gene expression by modifying the interaction of *ADA* pre-mRNA with, among others, PTBP1 (Polypyrimidine Tract Binding Protein 1) and SRSF3 (Serine and arginine Rich Splicing Factor 3), which are fine regulators of lymphocyte activation [18,19,20]. Such in silico prediction prompts us to evaluate the possible contribute of rs244072 to MS disease.

The genetic screening of a population of 561 patients with MS showed no significant departure from Hardy–Weinberg equilibrium in the study population (frequency of non-risk allele T = 91.35%; frequency of risk allele C = 8.65%; chi-square n.s. *p* = 0.238; Figure 1B). The genotype distribution was as follows: TT (*n* = 469; 83.60%), TC (*n* = 87; 15.51%), CC (*n* = 5; 0.89%). For further analyses, patients were grouped as: CT + CC (*n* = 92, 16.40%) or TT.

### 3.2. The ADA SNP rs244072 and Clinical Characteristics

The clinical and demographic characteristics of MS patients involved in the study are shown in Table 1.

A significant association emerged between the SNP rs244072 and clinical characteristics (Table 2).

**Figure 2:**
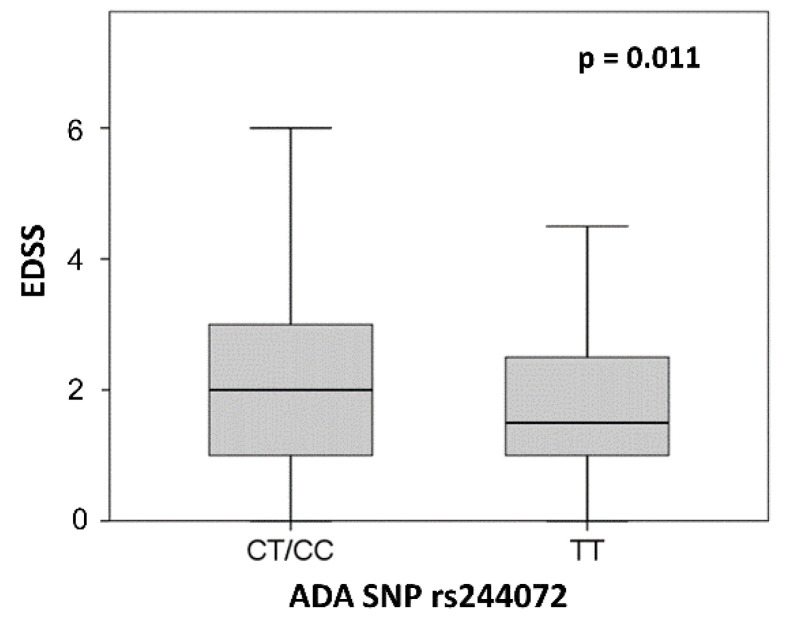
*ADA SNP rs244072* and EDSS. EDSS score according to SNP rs244072 genotype (CT/CC vs. TT). Mann–Whitney test *p* value is shown. Abbreviations: expanded disability status scale (EDSS), single nucleotide polymorphism (SNP).

In particular, the presence of the C allele was associated to higher EDSS at the time of diagnosis (TT patients median = 1.5, IQR = 1–2.5; CT/CC median = 2, IQR = 1–3; *p* = 0.011) (Figure 2). No other significant differences emerged between the two groups in demographic and other clinical characteristics examined at the time of LP.

### 3.3. The ADA SNP rs244072 and CNS Inflammation

**Figure 3:**
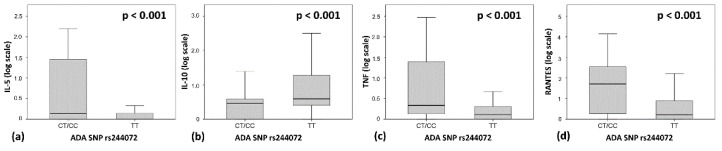
ADA SNP rs244072 and CSF inflammation. CSF concentrations of (**a**) RANTES, (**b**) TNF, (**c**) IL-5 and (**d**) IL-10 according to SNP rs244072 genotype (CT/CC vs. TT). To obtain a better graphical representation, CSF cytokines concentrations are shown in logarithmic scale. Mann–Whitney test p values after Benjamini–Hochberg correction are shown. Abbreviations: cerebrospinal fluid (CSF), tumor necrosis factor (TNF), interleukin (IL), single nucleotide polymorphism (SNP).

To explore whether individual genetic variability in *ADA* gene could influence central inflammation in MS, we studied the possible association between rs244072 alleles and the CSF levels of proinflammatory and anti-inflammatory molecules in 258 patients. As shown in Figure 3, we observed that patients with MS carrying the C allele, presented significantly higher CSF levels of TNF (TT patients median = 0.29, IQR = 0–1.03; CT/CC median = 1.15, IQR = 0.31–24.65; B-H adjusted *p* < 0.001), IL-5 (TT patients median = 0, IQR = 0–0.39; CT/CC median = 0.37, IQR = 0–27.33; B-H adjusted *p* < 0.001) and RANTES (TT patients median = 0.62, IQR = 0–6.72; CT/CC median = 49.97, IQR = 0.65–373.25; B-H adjusted *p* < 0.001). In addition, patients with the C allele showed lower levels of IL-10 (TT patients median = 2.9, IQR = 1.53–17.91; CT/CC median = 1.9, IQR = 0–2.83; B-H adjusted *p* < 0.001) in the CSF. These results indicate that the different genetic variants of *ADA SNP rs244072* could participate in determining the CNS inflammation in MS patients.

### 3.4. ADA SNP rs244072 Peripheral Blood Lymphocytes

We explored whether the *ADA* group (TT vs. CT/CC) was associated to WBC count, number of Neutrophils, Lymphocytes, and NLR, in an independent cohort of MS patients (*n* = 230; Sex, F = 151 (65.7%); MS Phenotype RR/CIS/RIS = 194 (84.3%); SP/PP = 36 (15.7%); Age, mean (SD) = 39.13 (12.52), EDSS, median (IQR) = 2 (1–3)). No significant differences emerged in the demographic and clinical characteristics of the two groups of MS patients.

**Figure 4:**
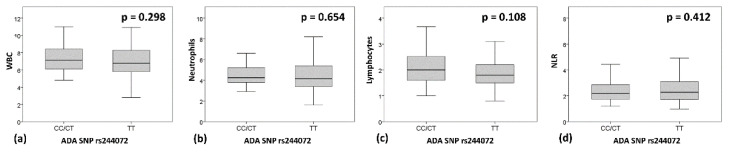
*ADA* SNP rs244072 and WBC, neutrophils and lymphocytes. Peripheral blood levels of (**a**) WBC, (**b**) neutrophils and (**c**) lymphocytes, and (**d**) NLR, according to SNP rs244072 genotype (CT/CC vs. TT). Mann–Whitney test p values are shown. Abbreviations: neutrophil/lymphocyte ratio (NLR), single nucleotide polymorphism (SNP), white blood cells count (WBC).

Although WBC and particularly lymphocyte were slightly reduced in the TT group (Figure 4), suggesting a reduced function of *ADA* enzyme, no statistically significant differences were found between the two groups (WBC, median (IQR): “TT” = 6.77 (5.8–8.28); “CT/CC” = 7.125 (6.07–8.49); *p* = 0.298. Neutrophils, median (IQR): “TT” = 4.15 (3.4–5.4); “CT/CC” = 4.25 (3.77–5.2); *p* = 0.654. Lymphocytes, median (IQR): “TT” = 1.8 (1.5–2.2); “CT/CC” = 2 (1.57–2.54); *p* = 0.108. NLR, median (IQR): “TT” = 2.28 (1.72–3.09); “CT/CC” = 2.17 (1.71–2.85); *p* = 0.412).

## 4. Discussion

Although *ADA* activity is critically involved in immune response homeostasis, the influence of *ADA* individual variability on MS course and CNS inflammation has not been investigated. In the present study, we found that *ADA* gene SNP rs244072 is associated with disability and with different CSF levels of specific proinflammatory and anti-inflammatory molecules at the time of diagnosis. Particularly, patients bearing at least one C allele showed both exacerbated CSF inflammation and higher EDSS compared to TT patients.

Different studies have demonstrated that CSF inflammatory milieu critically influences the clinical course of MS. Increased CSF levels of specific proinflammatory molecules at the time of diagnosis were found to be associated with enhanced prospective disease activity, disability and neurodegeneration [5]. Conversely, it has been shown that anti-inflammatory molecules and growth factors could beneficially influence MS progression, contrasting neuroinflammation [22] and promoting clinical stability [23].

Our results suggest that the association between individual variability of *ADA* gene and a proinflammatory CSF milieu may negatively influence the disease course of MS. In particular, the presence of a C allele of *ADA* gene SNP rs244072 is associated to enhanced expression of TNF, RANTES and IL-5, and reduced concentrations of anti-inflammatory molecules, such as IL-10. The role of these cytokines in the pathogenesis of MS has been clearly demonstrated both in preclinical and clinical studies.

TNF is a major proinflammatory cytokine with a crucial pathogenic role in MS [3,4,24], extensively investigated in humans and MS animal models. These studies clearly showed that TNF is able to promote synaptic hyperexcitability and excitotoxic neurodegeneration. Accordingly, the blockage of TNF signaling in the experimental autoimmune encephalomyelitis (EAE) mice ameliorated the clinical score, reducing both synaptic alterations and dendritic loss [24]. More recently, it has also been observed that T lymphocytes isolated from patients with RRMS during a relapse, presented high levels of TNF compared to healthy subjects or RRMS patients in the remitting phase. The incubation of relapsing RRMS T cells on murine brain slices of healthy mice triggered glutamatergic synaptic dysfunctions, mimicking EAE synaptic alterations and leading to excitotoxic damage, and a treatment with etanercept, a TNF receptor antagonist, was able to prevent the appearance of such alterations [4]. Moreover, high TNF levels have been associated to progressive disease course both in EAE and in MS [25]. Notably, CSF from patients with progressive MS induced a TNF-mediated increase in the excitatory transmission together with a glutamate-driven neuronal damage when incubated on murine brain slices of healthy mice. In this context, rituximab-induced B cell depletion was associated with a marked reduction in NF levels in the CSF, electrophysiological alterations and biomarkers of neuronal damage [25], further supporting the pivotal role of TNF in MS pathophysiology. Like TNF, RANTES (regulated upon activation normal T-cell expressed and secreted) is an inflammatory mediator involved in the pathogenesis of neuroinflammation in MS. RANTES promotes immune cells migration through the blood brain barrier, modulating the expression of adhesion molecules [26]. T lymphocytes derived from MS patients overexpress RANTES receptor CCR5 [27], and RANTES production is enhanced in peripheral blood mononuclear cells from patients with RRMS [28], and elevated RANTES CSF levels have been reported in relapsing patients [29]. The pathogenic role of this cytokine is further supported by the association between polymorphisms regulating the expression of CCR5 and MS severity [30].

Although we found an association between *ADA* SNP and IL-5 levels, the role of this cytokine in MS pathogenesis is uncertain. IL-5 is released by Th2 lymphocytes and is mainly involved in eosinophil differentiation, and its production was found to be increased by glatiramer acetate [31].

Our observation that C-carriers in rs244072 SNP present higher EDSS and lower CSF levels of the anti-inflammatory cytokine IL-10, is coherent with previous studies showing that IL-10 exerts several beneficial effects in EAE and MS. IL-10 induces regulatory T cells resulting in reduced microglia activation, less neuroinflammation and neurodegeneration [22]. Conversely, impaired IL-10 expression may sustain neuroinflammation, as the decreased expression of IL-10 mRNA has been reported in MS patients, and reduced levels of IL-10 have been associated with clinical activity and disease progression [32]. In MS patients, the suppression of CD4^+^ T cell proliferation, mediated by IL-10, was shown to be less effective [33].

The effect of different disease-modifying therapies (DMTs) may be mediated by the modulation of the inflammatory milieu. Treatment with IFN-B in MS is associated with reduced production of TNF compared to untreated patients [34], decreased RANTES levels [28], and increased IL-10 release in blood cell cultures [35]. Treatment with cladribine has been associated with a reduced relapse rate and decreased progression of disability [36]. In particular, cladribine reduced the release of inflammatory cytokines by human T lymphocytes in vitro [9] and treatment with cladribine tablets was associated with lower CSF and serum levels in the IL-8 and RANTES [37].

Our result suggests that the modulation of *ADA* activity could beneficially influence CSF cytokine milieu in MS, possibly reducing disability progression and neurodegeneration. Indeed, despite the fact that the functional consequences of the different rs244072 alleles are poorly understood, the SNP is an intronic variant with possible regulatory implications because it is included in the RNA binding site for important regulators of lymphocyte activation, such as PTBP1 and SRSF3. In combination with the results of the in silico analysis, the small decrease in lymphocyte count in TT patients may be explained by a reduction in *ADA* activity, possibly due to less efficient gene expression, justifying, at least in part, reduced central inflammation and disability in these patients compared to C-carriers. Additionally, its relevance for MS may be indirectly inferred by recent evidence that genes that are disrupted in Mendelian disorders (i.e., *ADA* deficiency) are dysregulated by noncoding variants in complex traits [38]. Furthermore, our results suggest that the same variant associates with both disease course and immunological parameters (TNF, RANTES and IL-5). This “coincident association” between disease and intermediate phenotypes is known to increase the interest in any development linked to the therapeutic modulation or monitoring of the intermediate phenotypes themselves [39,40]. These considerations warrant further investigations on how genetic variants of *ADA* affect the enzyme’s activity (together with the above immunological parameters) in MS patients and further studies exploring different SNPs of *ADA* gene are required to better characterize the relationship between *ADA* genetic variability and MS pathogenesis. It is also important to prospectively investigate MRI measures of neurodegeneration, and the association with CSF biomarkers of neuronal damage, including tau protein, neurofilaments, and β amyloid.

## 5. Conclusions

The present investigation highlights, for the first time, the potential role of genetic individual variability of ADA gene in regulating central inflammation and clinical manifestations in MS. These results deserve further investigation to better comprehend the possible mechanisms of action of cladribine tablet treatment in MS.

## Figures and Tables

**Table 1:** Demographic and clinical characteristics of MS patients.

		MS Patients *n* = 561
CIS	N (%)	91 (16.2)
RIS	N (%)	17 (3)
RR-MS	N (%)	404 (72)
SP/PP-MS	N (%)	49 (8.8)
Sex, F	N (%)	373 (66.5%)
Age, years	Mean, (SD)	36.1 (10.63)
Disease duration, months	Median (IQR)	8.17 (1.42–48.72)
EDSS at diagnosis	Median (IQR)	2 (1–2.5)
Clinical activity at LP	N (%)	215 (38.3)
Radiological activity at LP	N (%)	238 (42.4)

Abbreviations: female (F), multiple sclerosis (MS), relapsing-remitting (RR), clinically isolated syndrome (CIS), radiologically isolated syndrome (RIS), secondary-progressive (SP), primary-progressive (PP), expanded disability status scale (EDSS), interquartile range (IQR).

**Table 2:** Demographic and clinical characteristics of MS patients according to SNP rs244072 group.

		CT/CC *n* = 92 (16.4%)	TT *n* = 469 (83.6%)	*p* Value
Sex, F	N (%)	59 (64.1)	314 (67)	0.60
Age, years	Mean, (SD)	38.03 (11.99)	36.09 (11.01)	0.213
MS phenotype	RR/CIS/RIS, n (%)	85 (92.4)	427 (91)	0.676
	SP/PP, n (%)	7 (7.6)	42 (9)	
Disease duration, months	Median (IQR)	10.4 (1.62–48.17)	7.9 (1.42–49.70)	0.930
EDSS	Median (IQR)	2 (1–3)	1.5 (1–2.5)	0.011
Clinical activity at LP	N (%)	34 (37)	181 (38.6)	0.768
Radiological activity at LP	N (%)	42 (45.7)	196 (41.8)	0.493

Subjects carrying C allele of SNP rs244072 (CT/CC), TT homozygous subjects for SNP rs244072 (TT). Abbreviations: multiple sclerosis (MS), relapsing-remitting (RR), clinically isolated syndrome (CIS), secondary-progressive (SP), primary-progressive (PP), expanded disability status scale (EDSS), interquartile range (IQR), lumbar puncture (LP).

## Abbreviations

ADA	Adenosine deaminase
CIS	Clinically isolated syndrome
CNS	Central nervous system
CSF	Cerebrospinal fluid
EAE	Experimental autoimmune encephalomyelitis
EDSS	Expanded disability status score
Gd	Gadolinium
Gd+	Gadolinium-enhancing
IL	Interleukin
IQR	Interquartile range
LP	Lumbar puncture
MS	Multiple Sclerosis
NLR	Neutrophil/lymphocyte ratio
PP	Primary-progressive
PTBP1	Polypyrimidine Tract Binding Protein 1
RR	Relapsing-remitting
SP	Secondary-progressive
SRSF3	Serine and arginine Rich Splicing Factor 3
SNP	Single nucleotide polymorphism
SD	Standard deviation
TNF	Tumor necrosis factor
WBC	white blood cells
%	Frequency
